# Sensory and physicochemical profiling of traditional and enriched gari in Benin

**DOI:** 10.1002/fsn3.1201

**Published:** 2019-09-09

**Authors:** Laurent Adinsi, Noël Akissoé, Andres Escobar, Laure Prin, Nadège Kougblenou, Dominique Dufour, Djidjoho J. Hounhouigan, Geneviève Fliedel

**Affiliations:** ^1^ Faculté des Sciences Agronomiques Université d’Abomey‐Calavi Cotonou Benin; ^2^ CIAT Cali Colombia; ^3^ CIRAD UMR QUALISUD Montpellier France; ^4^ Qualisud Univ Montpellier CIRAD Montpellier SupAgro Univ d'Avignon Univ de La Réunion Montpellier France

**Keywords:** Cassava, Gari, palm oil, physicochemical properties, sensory profile, soybean

## Abstract

Gari is a roasted fermented granular product made from cassava in many African countries. It is consumed raw, or added with water, or cooked into a paste. Up to now, gari enriched with palm oil and/or soybean is not available on Beninese markets. To our knowledge, no sensory profiling using appropriate methodology has been conducted on gari in Benin. The sensory studies on gari in Benin and other African countries only included general descriptors (appearance, taste, odor). The aim of our study was to establish a detailed sensory and physicochemical profile of nine traditional and three enriched gari made using different processes in Benin. Fifteen sensory descriptors of raw gari, and gari added with water, were generated and scored using quantitative descriptive analysis. The enriched gari differed from traditional gari mainly in color and odor, while their swelling capacity, texture during chewing, and light sour taste were similar. Marked variability in particle size, particle heterogeneity, water absorption, and sour taste was found among traditional gari. The physicochemical characteristics, such as degree of starch gelatinization, L‐lactic acid, and β‐carotene contents, were highly variable among the 12 gari. Multifactor analysis revealed highly significant correlations between some physicochemical and sensory properties. The addition of soybean and/or palm oil did not affect most of the sensory properties of the traditional gari. The acceptability of these enriched gari with higher nutritive value by Beninese consumers should be tested to develop marketing strategies.

## 1 INTRODUCTION

Cassava (*Manihot esculenta* Crantz) production in Benin increased from 2.3 to 4.2 Mt between 2000 and 2016 (FAOSTAT, 2016). Gari, a roasted fermented granular product, is one of the most popular staple foods made from cassava and is consumed in several different forms in Benin (Agre et al., 2017). Gari is usually consumed raw (dry), or added with water, sugar, groundnuts, and/or cashew nuts, or cooked into a paste named Eba, or sprinkled on cooked cowpea beans. Gari is obtained from cassava roots after several successive steps: peeling, washing, grating, pressing/fermenting, sieving, and cooking/drying (Escobar et al., 2018). Marked variability is observed among traditional gari, depending on processes used which give the gari different sensory properties (color, particle size, dryness, and sourness). This variability has also been reported in Nigeria (Makanjuola, Ogunmodede, Makanjuola, & Awonorin, 2012) and Ghana (Oduro, Ellis, Dziedzoave, & Nimako‐Yeboah, 2000). Laya, Koubala, Kouninki, and Nukenine (2018) reported in Cameroon that the physicochemical and sensory characteristics of gari varied significantly with the variety and the harvest period. More recently, gari fortified with palm oil and/or soybean was developed in Benin by research and endogenous innovative actions, but up to now, these new types of gari are not available on Beninese markets, whereas they are readily available in Nigeria and their processes (Akinoso & Olatunde, 2014), physicochemical characteristics (Edem, Ayatse, & Itam, 2001; Karim, Balogun, Oyeyinka, & Abolade, 2015), and sensory properties (Osho, 2003; Sanni & Sobamiwa, 1994) are largely documented. In most Nigerian enriched gari, the ingredients (palm oil and/or soybean) are usually added to the fermented mash prior to roasting, whereas in Benin they are added in the mash before fermentation. Many studies have been conducted on sensory evaluation of cassava products including gari (Bechoff et al., 2018; Makanjuola et al., 2012; Oda, Ewa, Amah, Ejiofor, & Wassagwa, 2018; Sanoussi et al., 2015), fufu (Sobowale & Oyewole, 2008), and other root and tuber products (Abiodun & Akinoso, 2015). Most were conducted in Nigeria on a few general descriptors (appearance, taste, odor), using hedonic tests with a panel to determine the acceptability of improved products. Few studies undertook a complete sensory descriptive analysis to better characterize a product (Adinsi et al., 2015; Leighton, Schonfeldt, & Kruger, 2010; Muoki, Kinnear, Emmambux, & Kock, 2015; Nindjin et al., 2007). Our study aims to establish a sensory and physicochemical profile of nine traditional gari collected from various areas in Benin, and to position three enriched gari on this sensory map.

## 2 MATERIALS AND METHODS

### 2.1 Gari samples

**Figure 1:**
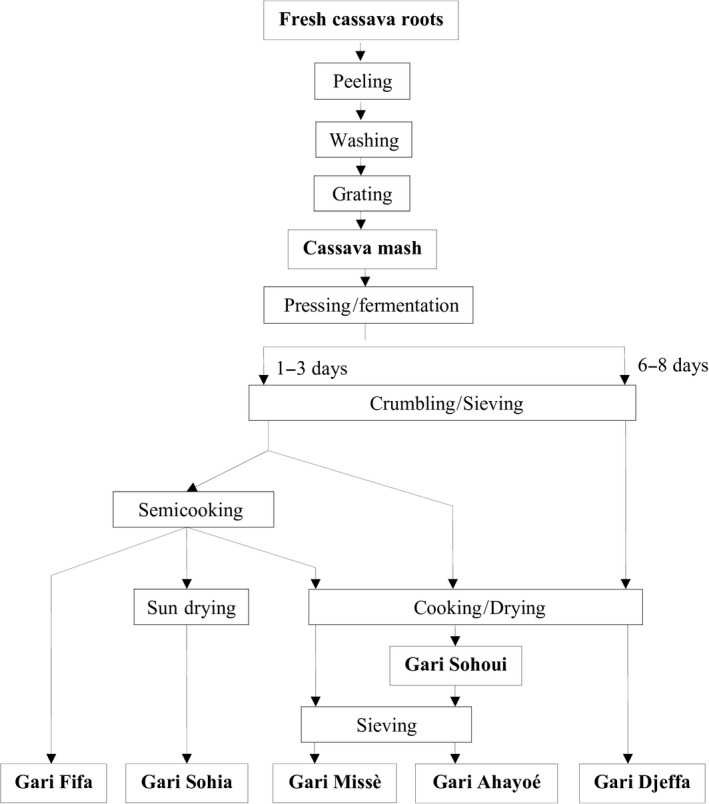
Flow diagram of cassava processing into various types of Beninese traditional gari

Nine traditional gari samples with different characteristics were collected from processors at different locations in the South, South‐East, and Center‐North regions of Benin (Table 1). The samples were produced using the different processes with different pressing/fermentation, cooking/drying, and sieving conditions, shown in the flow diagram in Figure 1. They belong to the six main traditional types of gari below:

- Gari Missè is produced in a specific area of Savalou district, named Missè, Center‐North region. It is made more carefully than elsewhere with two successive cooking steps in two separate earthenware pans called “*canari*,” one for cooking the product (to gelatinize the starch), the other to roast it until it is almost completely dry, while stirring with a spatula of wood. Gari Missè is sieved after roasting. It is well known for its high quality (cleaner, finer, and drier).
- Gari Sohoui is cooked in only one *canari* on a wood fire, until it is almost completely dry.
- Gari Ahayoé is a Sohoui‐type with a very fine particle size due to an additional sieving after cooking.
- Gari Sohia is a semidried gari. It is obtained with a partial cooking step in an iron roasting pan followed by a drying in the sun. The central part of the particles may be slightly wetter.
- Gari Fifa is partially cooked with no sun drying and no sieving. It is slightly wetter than the others.
- Gari Djeffa is Sohoui‐type gari from Djeffa, South region, between Cotonou and Porto‐Novo. Cassava mash is fermented in jute bags for 5 to 8 days according to an old custom. This gari is brownish‐orange in color and tastes extremely sour.

**Table 1:** Traditional and enriched gari samples collected in different regions in Benin

Gari	Ingredients added	Pressing fermentation (days)	Cooking (number of cooking, duration)	Village Region	Code
Missè		1	2; long	Savalou, Center‐North	*Missè.UF.Sa*
Sohoui		2–3	1; long	Ikpinlè, South‐East	*Sohoui.F.Ik*
Sohoui		2–3	1; long	Savalou, Center‐North	*Sohoui.F.Sa*
Ahayoé		1	1; long	Ikpinlè, South‐East	*Ahayoe.UF.Ik*
Ahayoé		1	1; long	Paouignan, Center‐North	*Ahayoe.UF.Pa*
Sohia		2–3	1 short + sun drying	Dassa, Center‐North	*Sohia.F.Da*
Sohia		1	1 short + sun drying	Dassa, Center‐North	*Sohia.UF.Da*
Fifa		1	1; short	Ikpinlè, South‐East	*Fifa.UF.Ik*
Djeffa		6–8	1; long	Djeffa, South	*Djeffa.F.Dj*
Soybean	Soybean paste	1	1; long	Ouedo, South	*Soy.UF.Ou*
Palm oil	Palm oil	1	1; long	Ouedo, South	*Palmoil.UF.Ou*
Soy–Palm oil	Soybean paste and Palm oil	1	1; long	Ouedo, South	*Soy–Palmoil.UF.Ou*

Three enriched gari supplemented with soybean and/or palm oil were obtained from a group of women processors in Ouèdo (South Benin). They made the new types of gari developed by the University of Abomey‐Calavi, Benin. These gari are still very scarce on the local markets. They were produced by using the flow diagram of a Sohoui‐type in which soybean paste and/or palm oil was added to the cassava mash before fermentation (4 kg/100 kg and/or 1 L/100 kg, respectively). Soybean paste was obtained after boiling the soybeans at 100°C for 45 min, dehulling the boiled soybeans by rubbing them between the hands to remove the coat, and crushing them into a paste using a millstone. Palm oil was purchased at a local market.

All the gari samples are labeled using three words: *gari type.F(or UF).location* where *F* means fermented and UF means unfermented (or rather fermented for a very short period of no more than one day, but considered by Beninese consumers as unfermented gari) (Table 1).

### 2.2 Ethical assessment and consent

The study was assessed and approved by the University of Abomey‐Calavi, Faculty of Agricultural Sciences, School of Nutrition and Food Sciences and Technology. The panelists participating in the study gave their consent. They were informed that their participation was entirely voluntary, and they could withdraw from the panel at any time.

### 2.3 Sensory descriptors

The sensory profile of gari samples was established by 18 semitrained panelists using a simplified quantitative descriptive analysis (QDA) with no standard provided (Meilgaard, Civille, & Carr, 2015; Tomlins, Owori, Bechoff, Menya, & Westby, 2012). The members of the sensory panel were selected for their familiarity with the product. Sessions were conducted at an air‐controlled temperature of 28°C. The language used for the sensory testing was French. No information on the gari samples was provided to the panel. During a preliminary focus group session facilitated by the panel leader, panelists were asked to generate sensory descriptors for eight different raw gari and sensory descriptors for the same eight gari added with water (gari/water ratio of 1/6) regarding their appearance, odor, texture, and taste. After eliminating similar terms, 15 sensory descriptors were chosen in consensus by the group of panelists, and drawn up on the score sheet: nine sensory descriptors for the description of raw gari and six for gari added with water. A clear definition of each descriptor and its scale end‐terms was established by the panel during previous round table sessions (Table 2).

**Table 2:** Definition of sensory descriptors of raw gari and gari added with water

	Sensory descriptors	Definition
Raw Gari		
Appearance	White	The color can range from white (pure white) to light brown (off‐white).
	Yellow	The color can range from a very pale yellow to a bright yellow.
	Particle size	The size of the majority of particles can range from small to coarse.
	Particle heterogeneity	The heterogeneity of particle size can range from slightly heterogeneous (most particles are the same size) to highly heterogeneous (most particles differ in size).
Odor	Odor of palm oil	The intensity of the odor of palm oil can range from no odor to strong odor.
	Odor of added ingredient	A particular odor, different from that of palm oil, due to the addition of an ingredient, may range from no odor to a strong odor.
Texture	Degree of drying	The degree of dryness perceived in the mouth, can range from low (the gari is not dry enough) to high (the gari is very dry).
	Texture during chewing	The texture can range from soft to crispy, with an intermediate texture: crispy on the outside of the particle and soft inside.
Taste	Sweet taste	The taste may range from not sweet to very sweet.
Gari added with water		
Appearance	Water cloudiness	The appearance of the water added to gari after a few seconds, can range from clear to cloudy.
	Water Absorption	The absorption of water, after 1–2 min, resulted in an increase of the volume of gari in the transparent plastic glass. It can range from a small to a large amount of water absorbed.
	Fibers on the water surface	After a few seconds of stirring, the amount of fibers on the surface of water can range from a few fibers to many fibers.
Odor	Fermented odor	The odor of gari can range from unfermented to very fermented.
Taste	Sour taste	The taste of gari may range from not sour to very sour.
	Bitter aftertaste	At the end of chewing, an aftertaste may be perceived ranging from not bitter to a strong bitter aftertaste.

### 2.4 Quantitative descriptive analysis (QDA)

The panelists first attended four training sessions on two consecutive days in how to clearly define each descriptor, use the scoring scale correctly, and to improve their ability to discriminate between descriptors. After the training, the 12 gari samples were tested in triplicate by the panel over the course of nine different sessions. At each session, four gari samples, coded with a random three‐digit number, were served to each panelist in random order, raw gari was served on a green plastic plate, and gari was added with water in a transparent plastic glass. After scoring the nine sensory descriptors of one raw gari, the panelists were asked to add water to the transparent plastic glass containing the same raw gari and to score the six sensory descriptors for gari added with water. In this way, the 15 sensory descriptors were scored successively for each of the four gari samples per session. The intensity of each descriptor was scored by putting a mark on a 100‐mm unstructured linear scale anchored at the ends (minimum rating at the left end to maximum rating at the right end). A glass of water was provided, so the panelists could rinse their mouth between tasting each sample.

### 2.5 Physicochemical analysis

Proximate analysis [moisture and crude fiber (CF)] were determined according to AOAC (2000) procedures.

The particle size profile was determined on 50 g of gari sample using a Ro‐Tap RX‐29‐E test sieve shaker (W.S. Tyler) operating with eight sieves (from 150 µm to 3,350 µm mesh) for 5 min. The mean particle size D50 was defined as the sieve mesh that retained 50% of the gari sample.

The pH, titratable acidity, and swelling capacity of gari were determined by using specific methods (Bainbridge, Tomlins, Wellings, & Westby, 1996). Titratable acidity was defined as equivalent H^+^ per 100 g gari (d.b.). Swelling capacity was determined by the ratio of the volume of gari in the water to the initial volume of gari.

Starch content (g/100 g d.b.) was determined as the difference between total carbohydrate and free glucose contents. Total carbohydrates were evaluated after incubating the gari samples with Termamyl 120L (a heat‐thermostable *α*‐amylase enzyme) at 98°C, then with amyloglucosidase at 60°C, and measuring glucose release by spectrophotometry at 510 nm after reaction with the GOD‐POD enzymatic system at 35°C (Giraldo Toro et al., 2015).

The degree of starch gelatinization was measured using a differential scanning calorimeter (DSC 8500, Perkin Elmer). The enthalpy change due to gelatinization was assessed on gari samples. The ratio of starch gelatinization enthalpy after cooking (∆*H*_gari_) to before cooking (∆*H*_cassava mash_) was used to calculate the percent of gelatinized starch (% G) in the gari samples (Vidal et al., 2007):

$$\%G=\left[1-\left(\Delta H_{\text{gari}}/\Delta H_{\text{cassava mash}}\right)\right]\times 100.$$

Organic acids were determined by HPLC (Sanchez et al., 2013) using a lactic acid standard curve prepared with lactic acid (Sigma–Aldrich (85+%) 252,476).

Neutral detergent fiber (NDF), acid detergent fiber (ADF), and acid detergent lignin (ADL) were determined using the appropriate procedures (Van Soest, Robertson, & Lewis, 1991). Hemicellulose was expressed as the difference between NDF and ADF, and cellulose was defined as the difference between ADF and Lignin.

Hydrocyanic acid (HCN) content was determined using the appropriate method (Essers, Bosveld, Grift, & Voragen, 1993).

Total carotenoids were extracted (Ceballos et al., 2012), then separated, and quantified using a high performance liquid chromatography with a C30 reverse phase column and a DAD detector at 450 nm (Ceballos et al., 2012). Each carotenoid was quantified by integration of peak area, and its concentration was determined on a fresh weight basis by comparison with respective standard curves (Ceballos et al., 2012).

### 2.6 Statistical analysis

Principal component analysis was performed on the mean sensory scores of raw gari and gari added with water. Multifactorial analysis was performed to identify the relationships between sensory descriptors and physicochemical data. All analyses were computed using XLSTAT (Addinsoft, version 2013).

## 3 RESULTS AND DISCUSSION

### 3.1 Sensory profile of the nine traditional and the three enriched gari

**Figure 2:**
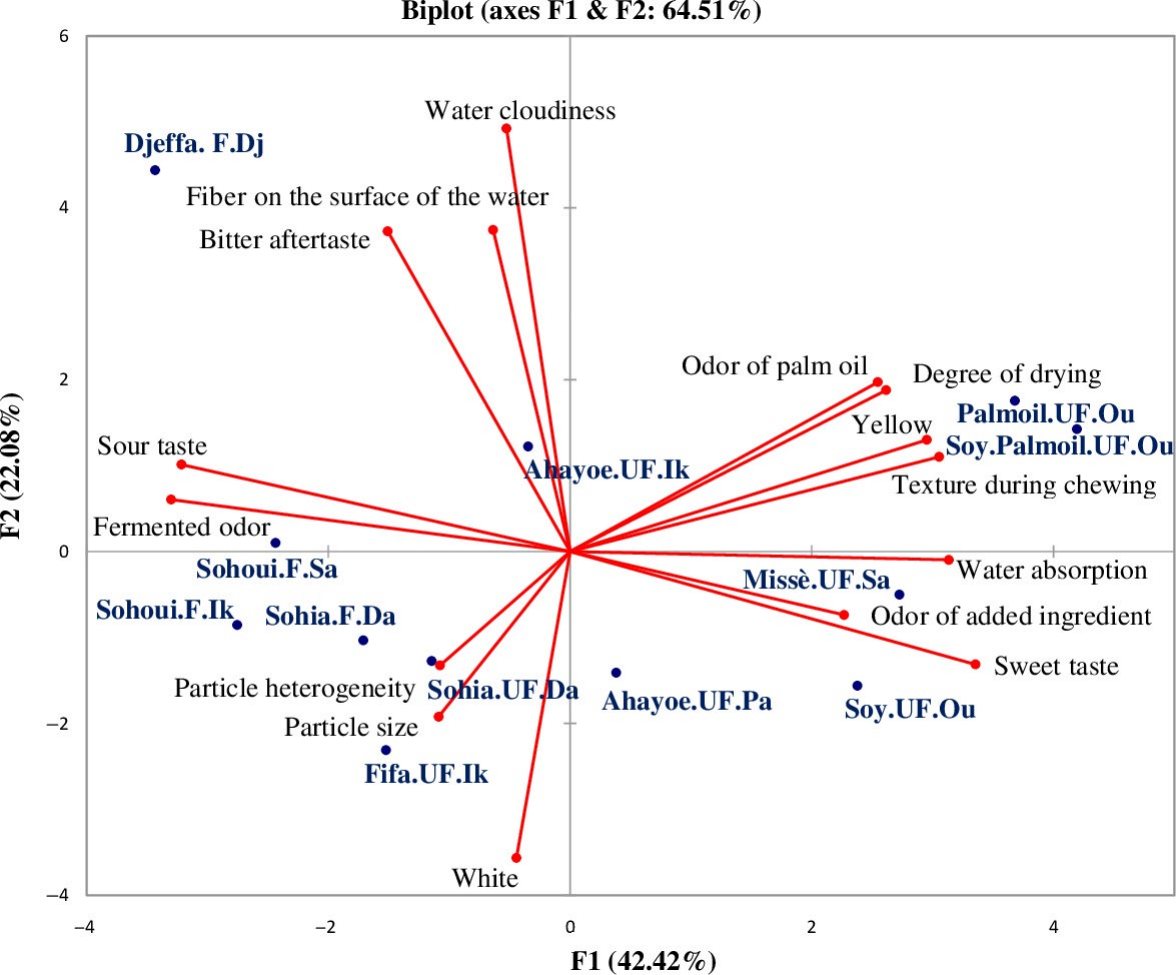
Sensory profile of traditional and enriched Beninese gari

Principal component analysis (PCA) was used to summarize the relationships between the 12 gari samples and the average intensity of the 15 sensory descriptors scored by the panelists. The PCA plot in Figure 2, explained 64.5% of the variance, the first and second axes accounting for 42.4% and 22.1%, respectively, and up to 92.1% when the third and fourth axes were also considered (Figure not shown). Axis 1 was mainly explained by the descriptors Yellow, Odor of palm oil, Degree of drying, Texture during chewing, Sweet taste, Water absorption, Fermented odor, and Sour taste. The enriched gari Palmoil.UF.Ou and Soy.Palmoil.UF.Ou were positively associated with the descriptors Yellow, Odor of palm oil, Degree of drying, Texture during chewing, Sweet taste, and Water absorption, while the descriptors Fermented odor and Sour taste were associated with the gari fermented for 2–3 days: Sohui.F.Sa, Sohui.F.Ik, and Sohui.F.Da. Axis 2 was mainly explained by the descriptors Water cloudiness, Fibers on the surface of the water, and Bitter aftertaste, and they were associated with Djeffa.F.Dj. These three descriptors described Djeffa.F.Dj better than Fermented odor or Sour taste, despite its longer period of fermentation. Negatively, Axis 2 was explained by the descriptor White associated with Ahayoe.UF.Pa, Sohia.UF.Da, and Fifa.UF.Ik. Axis 3, which accounted for 19.3% of the variance, was explained by the descriptors Particle size and Particle heterogeneity. These two descriptors were negatively related with Missè.UF.Sa and Ahayoe.UF.Ik. These two gari were described as the gari with the finest and the most homogeneous particles. Soy.UF.Ou was situated apart on the PCA plot, associated with the descriptor Odor of added ingredient, which contributed 30% to Axis 4.

Pearson's correlation coefficients were calculated to investigate relationships among sensory properties. Positive correlations were found between Degree of drying and Texture during chewing (0.99) and Water absorption (0.63). The sensory descriptors Sour taste and Fermented odor were positively correlated (0.96). Water cloudiness was positively correlated with the descriptors Bitter aftertaste (0.81) and Fibers on the surface of the water (0.75). Particle size was highly positively correlated with Particle heterogeneity (0.99).

### 3.2 Physicochemical properties of the 12 traditional and enriched gari

The moisture content of all the gari samples ranged from 1.8% (Missè.UF.Sa) to 10.0% (Fifa.UF.Ik) dry basis (Table 3). The average moisture contents of traditional gari collected in different processing centers in West Africa were between 4% and 18% (Aiyelegun, Owolarafe, Ogunsina, & Samuel, 2017; Felber, Azouma, & Reppich, 2017; Oduro et al., 2000). To our knowledge, a moisture content as low as that of gari Missè.UF.Sa has never been reported to date. Such low moisture content could be explained by the precision and time spent by processors during the cooking process, which is achieved in two successive steps in two separated earthenware *canari*: one for cooking the product on a low burning wood fire and the other for roasting it until it is almost complete dry on a hotter fire.

**Table 3:** Physicochemical properties of nine traditional and three enriched gari samples (dry basis)

Gari samples	Moisture (%)	Particle size D50 (µm)	Swelling capacity	Starch (%)	% Gelatinized starch	pH	Titratable acidity (%)	L‐Lactic (mg/g)	Crude fibers (%)	Lignin (%)	Cellulose (%)	Hemi cellulose (%)	HCN (ppm)
Missè.UF.Sa	1.8	439	4.0	79.2	82.9	4.3	0.8	10.0	3.3	1.3	1.6	10.5	9.8
Sohoui.F.Ik	10.0	774	2.8	64.6	94.6	4.0	1.7	25.4	3.3	0.5	2.4	9.9	19.8
Sohoui.F.Sa	9.1	702	3.3	75.2	91.7	4.3	1.5	26.0	3.9	0.3	3.0	13.5	7.7
Ahayoe.UF.Ik	5.9	524	3.3	82.3	89.6	4.0	0.7	8.7	3.7	0.3	2.6	8.6	13.5
Ahayoe.UF.Pa	7.7	513	3.2	82.7	90.1	4.4	0.9	17.9	3.5	0.3	3.0	9.4	4.6
Sohia.F.Da	8.3	1,145	3.3	75.2	94.4	4.2	1.6	25.2	3.3	0.4	3.1	10.9	9
Sohia.UF.Da	6.9	910	3.2	77.2	97.0	4.1	1.3	22.1	3.4	0.2	2.5	14.7	28.1
Fifa.UF.Ik	9.8	999	3.1	81.7	94.4	4.3	0.8	12.0	3.1	0.5	2.1	25.0	29
Djeffa.F.Dj	5.4	458	3.3	68.8	86.6	4.1	3.2	23.3	3.6	0.2	2.8	10.5	6.1
Soy.UF.Ou	5.1	932	3.7	74.3	90.8	5.4	0.9	6.4	6.1	0.6	3.9	25.8	9.3
Palmoil.UF.Ou	4.2	693	3.8	78.2	97.0	5.5	0.8	5.9	5.5	0.7	3.3	9.1	1.9
Soy–Palmoil.UF.Ou	4.1	690	3.6	79.9	97.3	5.4	0.8	5.7	5.1	0.8	2.6	14.2	1.5

The average particle size D50 ranged from 439 µm (Missè.UF.Sa) to 1,145 µm (Sohia.F.Da) (Table 3). The finest samples were Missè and Ahayoe types which were sieved after cooking, while the coarsest were Sohia and Fifa types which were not sieved after cooking. Other authors (Oduro et al., 2000) reported an average particle size of 0.63 to 1.02 mm in 13 samples collected from processors in three regions in Ghana.

Regarding swelling capacity, Sohoui.F.Ik had the lowest value (2.8) and Missè.UF.Sa the highest (4.0) (Table 3). All the gari samples had a good swelling capacity of more than 3 times their initial volume (Ajibola, Makanjuola, & Almazan, 1987). The three enriched gari had high swelling capacities (3.6–3.8) close to that of Missè.UF.Sa. A similar range was found in 14 samples collected in South Togo (Felber et al., 2017) and in the 13 samples collected in Ghana mentioned above (3.2–4.3 and 2.9–3.6, respectively) (Oduro et al., 2000). A lower swelling capacity (2.0–2.8) was reported for 59 gari samples collected in five states in South‐West Nigeria (Aiyelegun et al., 2017).

The starch content measured in the Beninese samples ranged from 64.6% to 82.7% (Table 3). Afoakwa, Kongor, Annor, and Adjonu (2010) who studied the effect of fermentation time (0–48 hr) reported starch contents of 60.5%–69.8% in traditional gari and of 58.4%–67.5% in the 10%‐soy–gari. Lower starch contents (42.3%–48.4%, dry basis) were measured in traditional *attieke* products collected in three regions of Southern Côte d’Ivoire (Djeni, N'Guessan, Toka, Kouame, & Dje, 2011), while starch rates of 80.2%–91.4% (dry basis) were reported in *attieke* made from four traditional and improved cassava varieties (Assanvo et al., 2017). These differences could be due to the different methods of analysis used or, as underlined by some authors (Assanvo et al., 2017), to the uncompleted release of sugars due to limited hydrolysis of starch by enzymes during fermentation, resulting in higher starch contents.

Starch in the 12 Beninese gari appeared to be partially gelatinized in all the samples analyzed. The degree of gelatinization ranged from 82.9% to 97.3% (Table 3). Tivana, Dejmek, and Bergenstahl (2013) found the percentage of gelatinized starch to be over 82% in several commercial and fortified gari.

The pH of the nine traditional Beninese gari ranged between 4.0 and 4.4 (Table 3). These values are close to those observed in gari collected from processing centers or markets in Nigeria and Ghana (3.6–4.9) (Oduro et al., 2000; Sanni, Adebowale, Awoyale, & Fetuga, 2008). The three enriched gari had a higher pH (5.4–5.5) than the traditional ones (Table 3). Adding an ingredient might make the gari less sour by dilution effect, and/or by buffering effect, mainly caused by soy proteins that slows down the pH drop, resulting in higher pH. Higher pH values were also reported for gari fortified with 13% soybean flour (4.96 compared to 4.79 for the control; Ahaotu et al., 2017), while a slight decrease in pH was found between unfortified and 10%‐soy–gari after 24 hr and 48 hr of fermentation (Afoakwa et al., 2010).

Titratable acidity ranged from 0.7% to 3.2%, with 0.7% to 1.3% for unfermented gari (1 day) including the three enriched gari, 1.5% to 1.7% for fermented gari (2–3 days) and 3.2% for Djeffa.F.Dj, the highly fermented gari (Table 3). The higher value may be due to the activity of lactic acid bacteria during the longer (8 days) fermentation process, which led to the production of more organic acids and other metabolites (Aiyelegun et al., 2017).

Lactic acid was the main organic acid in both the traditional and enriched gari samples, contents ranging from 5.7 to 22.1 mg/g in the unfermented group including the three enriched gari, and from 23.3 to 26.0 mg/g in the fermented group including Djeffa.F.Dj (Table 3). A positive correlation was found between titratable acidity and lactic acid content (0.69), in agreement with the results of a previous study (Djeni et al., 2011).

The main fiber fraction identified in the traditional and enriched Beninese gari samples was hemicellulose (8.6%–25.8%), followed by cellulose (1.6%–3.9%) and lignin (0.2%–1.3%) corresponding to a total fiber content (NDF) of 11.5%–30.3% (Table 3). The highest hemicellulose and NDF contents were found in the soybean enriched gari (Soy.UF.Ou) but also in the traditional Fifa.UF.Ik. Crude fiber content, mainly composed of cellulose and lignin, ranged from 3.1% to 6.1% in the 12 gari samples. Crude fiber contents of 0.27%–5.14% were reported in gari samples collected in processing centers in five states in South‐West Nigeria (Aiyelegun et al., 2017). Data variability may be due to the raw material and processing operations such as peeling, rasping, crushing, and sieving before and after cooking.

High variability in cyanide content was observed among the 12 Beninese gari samples (1.5 to 28.1 mg HCN/kg d.b.) (Table 3). Eight Beninese gari samples including the three enriched gari had cyanide contents of less than 10 mg/kg of HCN, the WHO recommended safe level (FAO/WHO, 1991), while four traditional gari had higher cyanide contents (13.5–29 mg/kg). The variability in cyanide contents of the final product probably depends on the cassava varieties but mainly depends on the processing. Agbor‐Egbe and Mbome (2006) showed that processing of cassava roots was an effective way to reduce cyanogen contents (197.3–951.5 mg/kg) to low levels (1.1–27.5 mg/kg) during the production of some Cameroonian foods (*bâton de manioc*, fufu and gari). The lowest values were measured in the two palm oil enriched gari, with 1.5 and 1.9 mg HCN/kg d.b. for Soy.Palmoil.UF.Ou and Palmoil.UF.Ou, respectively. These results may be explained by the formation of complexes between palm oil natural components (probably beta‐carotene, triglycerides) and CN^‐^, which cannot be estimated using the current methods of cyanide quantification (Uvere, 1999).

Carotenoid content was evaluated in the two gari samples enriched with palm oil. Average trans‐β‐carotene contents were 1.37 µg/g w.b. in both gari and cis‐β‐carotene (9‐cis, 13‐cis, and 15‐cis‐β‐carotene) contents were 0.84 and 0.88 µg/g w.b. in Palmoil.UF.Ou and Soy.Palmoil.UF.Ou, respectively. No α‐carotene was detected in the palm oil gari samples. In Nigeria, Oluba, Oredokun‐Lache, and Odutuga (2018) reported five times higher trans‐β‐carotene content in gari made from vitamin A‐biofortified yellow cassava. Bechoff et al. (2015) reported four times higher trans‐β‐carotene and cis‐β‐carotene (5.6 µg/g and 4.7 µg/g w.b., respectively) and α‐carotene content of 3.1 µg/g w.b. in similar conditions to the Beninese enriched gari, when palm oil was added to white cassava mash before fermentation. This difference may be due to a slightly higher amount of palm oil added (1.4 L/100 kg instead of 0.66 L/100 kg) but also to the composition of the palm oil. Trans‐β‐carotene contents reported by Gouado, Mawamba, Ouambo, Some, and Felicite (2008) were more than 200 times higher (309.7 µg/g) in laboratory gari samples added with the same quantity of palm oil (2 ml for 210 g of root, i.e., 1 L/100 kg) and 100 times greater in commercial gari samples. However, in that process, palm oil was added to the fermented partially dried gari just before frying at 120°C for 10 min. In those conditions, one can assume that the carotenoid content reported in the study (Gouado et al., 2008) was more that of palm oil than that of gari.

Pearson's correlation coefficients were calculated to investigate relationships among physicochemical properties. Negative correlations were found between moisture content and swelling capacity (−0.89), between titratable acidity and starch (−0.74), and between pH and L‐lactic acid (−0.59).

### 3.3 Relationships between sensory and physicochemical properties of the Beninese gari

**Figure 3:**
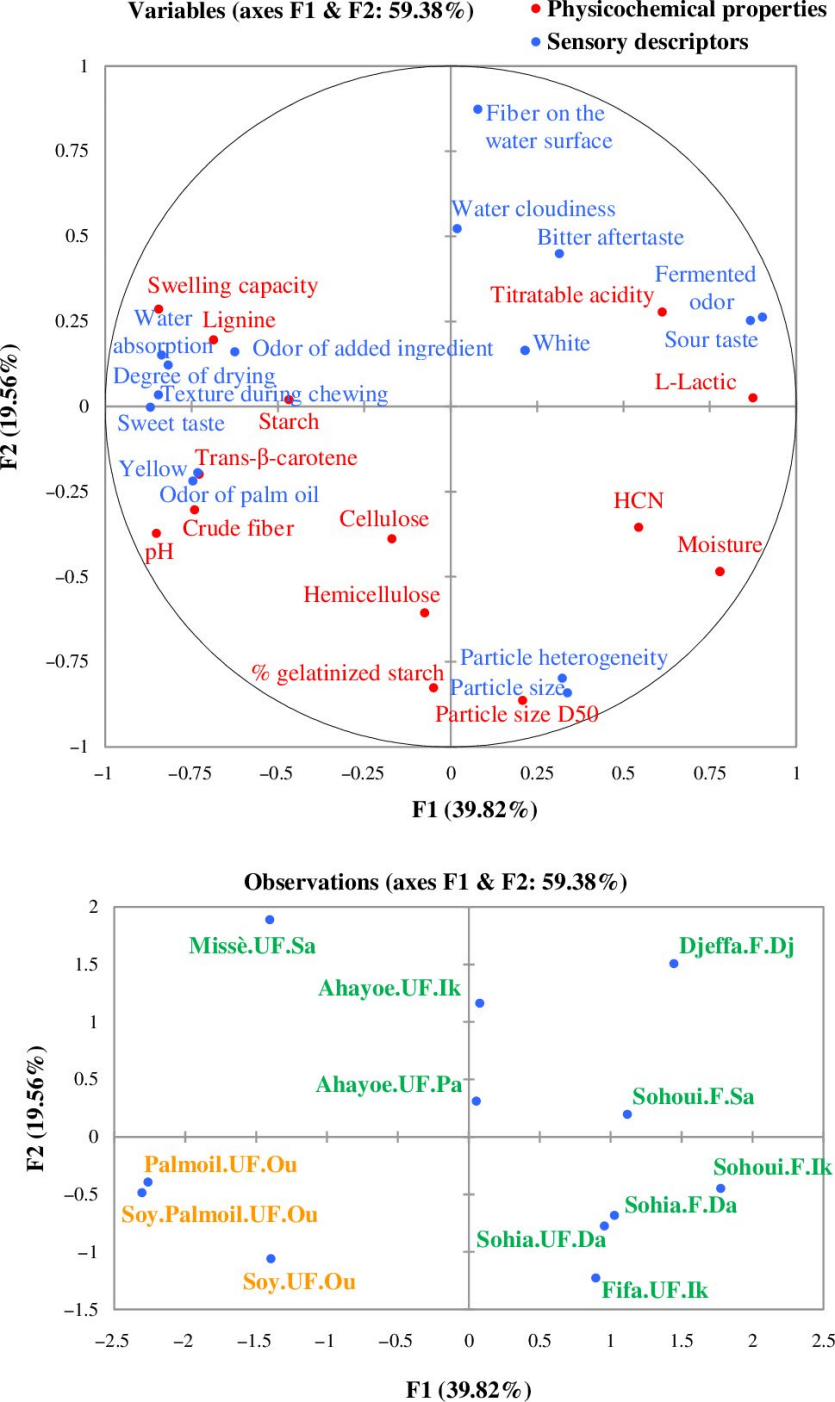
Multifactorial analysis of sensory and physicochemical properties of traditional and enriched Beninese gari

The relationships between physicochemical data and the mean scores of the 15 sensory descriptors for the 12 gari samples were analyzed by multi factorial analysis (MFA) (Figure 3). The MFA biplot explained 59.4% of the variance. Sour taste and Fermented odor were positively correlated with the physicochemical properties L‐lactic acid (0.94 and 0.92 respectively) and negatively with pH (−0.76 and −0.85). Similar correlations were reported for *attieke* collected in processing centers in Côte d’Ivoire (Djeni et al., 2011). These four characteristics were associated with Sohui.F.Ik, Sohui.F.Sa, and Sohia.F.Da. Sohia.UF.Da, which was described by the processors as unfermented (1 day), had L‐lactic and titratable acidity values close to those of fermented gari. The longer fermented and brownish gari, Djeffa.F.Dj, was highly associated with titratable acidity and with the descriptors Water cloudiness and Bitter aftertaste. The partially cooked and nonsieved gari types, Sohia and Fifa, were characterized by a high moisture content, and a high D50 particle size, which was positively correlated with the descriptors Particle size and Particle heterogeneity (0.86 and 0.80, respectively). Conversely, Missè.UF.Sa and Ahayoe.UF.Ik, which were sieved after cooking, were negatively associated with these three characteristics. The two gari enriched with palm oil were characterized by the descriptors Yellow and Odor of palm oil, both highly positively correlated with the Trans‐β‐carotene content (0.98). The three sensory descriptors Degree of drying, Texture during chewing and Water absorption were correlated with Swelling capacity (0.77, 0.77, and 0.73, respectively) and negatively with Moisture content (−0.83, −0.79, and −0.72). The two gari enriched with palm oil were associated with these five characteristics, similarly to Missè.UF.Sa and Soy.UF.Ou. The latter was mainly associated with the descriptor Odor of added ingredient.

All the traditional gari were attributed to four separate groups with respect to their contribution to Axis 1 (positively), Axis 2 (positively and negatively), and Axis 3 (negatively).

## 4 CONCLUSION

The sensory and physicochemical profiles of different types of Beninese gari collected from different processors are well documented here for the first time. Three enriched gari recently developed by research and not currently available on the market in Benin were perceived by the panelists as being different from the traditional ones, mainly in color and odor. Nevertheless, their Swelling capacity, Texture during chewing, and their light Sour taste appeared to be close to those of traditional gari. Further testing with a large number of Beninese consumers will be useful to assess the acceptability of these new types of gari and to propose marketing strategies for enriched gari with higher nutritive value in Benin.

## CONFLICT OF INTEREST

The authors declare that they do not have any conflict of interest.

## ETHICAL APPROVAL

The study was approved by the University of Abomey‐Calavi, Faculty of Agricultural Sciences, School of Nutrition and Food Sciences and Technology.

## INFORMED CONSENT

Written informed consent was obtained from all study participants.
